# MRI findings and clinical testing for preoperative diagnosis of long head of the biceps pathology

**DOI:** 10.1002/jeo2.70050

**Published:** 2024-10-15

**Authors:** David Gallinet, Maxime Antoni, ReSurg, Chinyelum Agu, Floris van Rooij, Mo Saffarini, Julien Berhouet, Christophe Charousset, Jacques Guery

**Affiliations:** ^1^ Clinique Saint Vincent ELSAN Besançon France; ^2^ Centre Epaule Main Besançon Besançon France; ^3^ SoFEC – French Shoulder and Elbow Society Paris France; ^4^ Clinique de l'Orangerie, ELSAN Strasbourg France; ^5^ ReSurg SA Nyon Switzerland; ^6^ Orthopaedic and Traumatologic Surgery University Hospital Trousseau of Tours Chambray les Tours France; ^7^ Service Orthopédie, Clinique de Turin Paris France; ^8^ Polyclinique du Val de Loire ELSAN Nevers France; ^9^ ReSurg SA Nyon Switzerland

**Keywords:** clinical tests, long head of biceps, magnetic resonance imaging, rotator cuff, tendinopathy

## Abstract

**Purpose:**

Determine whether combining magnetic resonance imaging (MRI) observations and clinical tests could substantially improve sensitivity for diagnosis of long head of the biceps tendon (LHBT) pathology.

**Methods:**

The authors retrospectively assessed a consecutive series of 140 patients who underwent arthroscopic rotator cuff repair for isolated supraspinatus tears. The presence of LHBT pathology was assessed preoperatively on MRI using three criteria and four clinical tests specific to shoulder injuries. Binary outcomes of MRI observations and four clinical tests were combined to identify combinations resulting in the best sensitivity using intra‐operative arthroscopic findings as reference.

**Results:**

The study cohort comprised 100 shoulders (58 men and 42 women) aged 56.6 ± 9.4 years (range, 30–76) at index surgery. A total of 29 combinations were tested to obtain the best diagnostic algorithm for LHBT pathologies. Only four combinations reached a sensitivity ≥0.75, but had a specificity <0.45. The ‘Speed or Signal’ combination achieved the highest sensitivity (Se: 0.88; 95% confidence interval [CI]: 0.73%–0.96%; Sp: 0.20; 95% CI: 0.10%–0.33%).

**Conclusion:**

The most important findings of this study were that, for the diagnosis of LHBT pathology using clinical tests alone, the Speed test had the highest sensitivity (Se, 0.74), and using MRI observations alone, the signal intensity had the highest sensitivity (Se, 0.68). Combination of ‘Speed test or Signal intensity’ substantially improved the sensitivity (Se, 0.88) but yielded the lowest specificity (Sp, 0.20). The clinical relevance of these findings is that using the combination ‘Speed or Signal’ for preoperative diagnosis, 88% of pathologic LHBTs would be correctly diagnosed, while 80% of healthy LHBTs could be misdiagnosed as pathologic.

**Level of Evidence:**

Diagnostic study, Level IV.

**Abbreviations**
CTA: computed tomography arthrography
FN: false negative
FP: false positive
LHBT: long head of the biceps tendon
MRI: magnetic resonance imaging
NPV: negative predictive value
PPV: positive predictive value
RCR: rotator cuff repair
RCT: rotator cuff tear
Se: sensitivity
Sp: specificity
TN: true negative
TP: true positive

## INTRODUCTION

When repairing rotator cuff tears (RCTs), the choice of whether or not to perform tenodesis or tenotomy of the long head of the biceps tendon (LHBT) is controversial [12, 19]. While some surgeons recommend systematic tenodesis or tenotomy regardless whether the LHBT is normal or pathologic [2, 16, 29], other surgeons suggest that tenodesis or tenotomy should only be performed in shoulders with LHBT pathology [4, 13, 15, 18, 21, 28, 30].

There is no clear evidence to support systematic LHBT procedures during rotator cuff repair (RCR). Conservation of a pathologic LHBT could seriously compromise clinical and functional outcomes, while tenodesis or tenotomy of a healthy LHBT can unnecessarily extend surgery time, cause postoperative pain, or result in the undesirable Popeye sign, negatively affecting the patients' quality of life [3, 26]. Therefore, preoperative diagnosis of the biceps should maximise sensitivity (reliability at detecting/ruling‐in pathology), even if it compromises specificity (reliability at eliminating/ruling‐out pathology) [17]. Two recent systematic reviews on diagnosis of LHBT pathology revealed that magnetic resonance imaging (MRI) has low sensitivity (range, 0.52–0.56) but high specificity (range, 0.64–0.99), while clinical tests have moderate sensitivity (range, 0.17–0.71) and specificity (range, 0.38–0.92) [11, 23]. Even though studies have been published on the diagnostic accuracy of either MRI or clinical tests, to the authors' knowledge, there are no published studies that attempted to improve the sensitivity or specificity of diagnosis of LHBT pathology, using combinations of MRI observations and clinical tests.

The purpose of the present study was therefore to determine whether combining MRI observations and clinical tests could substantially improve sensitivity for diagnosis of LHBT pathology (compared to MRI alone and clinical tests alone). The hypothesis was that a combination of MRI observations and clinical tests would grant higher sensitivity compared to MRI observations alone or clinical tests alone.

## METHODS

**Figure 1:**
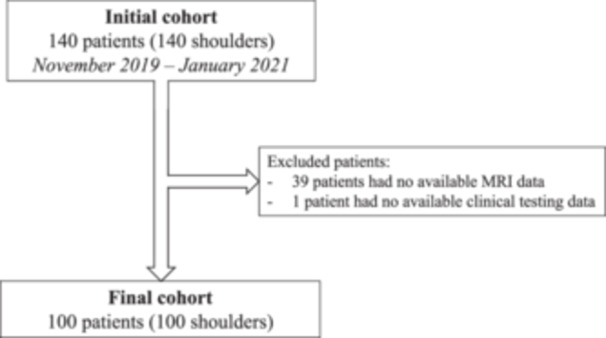
Flowchart following PRISMA guidelines.

The authors retrospectively included a consecutive series of 140 patients that underwent arthroscopic RCR for isolated supraspinatus tears, by eight orthopaedic surgeons at eight centres, between November 2019 and January 2021. Patients were excluded if they did not undergo preoperative MRI assessment (*n* = 39), or clinical testing (*n* = 1) (Figure 1). This left a study cohort of 100 patients in which biceps status was confirmed intra‐operatively during arthroscopy (considered as the ‘gold‐standard’ diagnosis).

### Magnetic resonance imaging

The presence of LHBT pathology was assessed preoperatively on MRI by each surgeon using three binary observations: (i) thickening of diameter, (ii) subluxation or dislocation from the bicipital groove and (iii) presence of signal intensity (T2 hypersignal).

### Clinical assessment

Patients were preoperatively assessed using four clinical tests specific to shoulder injuries: (i) Speed test [7], (ii) Yergason test [8], (iii) Kibler test [6] and (iv) bicipital groove tenderness. Each test rendered a binary outcome, positive in case of pain and therefore suspected pathologic LHBT, or negative if no pain was reported. Furthermore, the constant score and subjective shoulder value (SSV) were recorded following surgery.

### Combining MRI observations with clinical tests

Considering intra‐operative arthroscopic findings as the ‘gold‐standard’, the authors tested various combinations of binary MRI observations and binary clinical test results, to identify the combination that renders the highest sensitivity. The numbers of true positives (TPs), true negatives (TNs), false positives (FPs) and false negatives (FNs) were calculated for 29 combinations of MRI observations and/or clinical tests (18 sets of two criteria, 10 sets of three criteria and 1 set of four criteria).

### Ethical approval

All patients provided informed consent, and the study was approved by the local ethics committee (IRB:2018‐A01382‐53).

### Statistical analysis

Descriptive statistics were used to summarise the data. The sensitivity, specificity, accuracy, positive predictive value (PPV) and negative predictive value (NPV) were calculated for each of the 29 combinations of MRI observations and/or clinical tests. Statistical analyses were performed using R version 4.2.3 (R Foundation for Statistical Computing).

## RESULTS

The study cohort comprised 100 shoulders (58 men and 42 women) aged 56.6 ± 9.4 years (range, 30–76) at index surgery, with a body mass index of 26.6 ± 4.4 (range, 18.4–42.7). Of the 100 patients, 71 were operated on their dominant shoulder (71%), and 17 were smokers (17%). Patients had a mean preoperative Constant score of 56.0 ± 12.7, and a mean preoperative SSV score of 51.7 ± 13.3 (Table 1).

**Table 1:** Preoperative data.

	Final cohort (*n* = 100)	
	Mean ± SD	
	*N* (%)	Range
Constant score	56.0 ± 12.7	21–80
SSV	51.7 ± 13.3	20–80
Range of motion		
Passive forward elevation	171 ± 17.0	90–180
Passive abduction	153 ± 24.3	90–180
Passive external rotation 1	59 ± 13.7	30–90
Passive external rotation 2	83 ± 11.4	35–90
Active forward elevation	152 ± 29.2	50–180
Active abduction	137 ± 31.8	50–180
Active external rotation 1	50 ± 16.0	10–80
Active external rotation 2	78 ± 14.7	30–90
Active internal rotation		
(0) Grand trochanter	1	1%
(2) Buttock	10	10%
(4) Sacrum	5	5%
(6) L3	26	26%
(8) T12	32	32%
(10) T7	22	22%
C7	1	1%

*Note*: External rotation 1, arm at side and elbow at 90° flexion; External rotation 2, arm at 90° abduction and elbow at 90° flexion.

Abbreviations: C7, cervical vertabra 7; L3, lumbar vertabra 3; N, cohort size; SD, standard deviation; SSV, subjective shoulder value; T7, thoracic vertabra 7; T12, thoracic vertabra 12.

### Single criterion tests

Clinical testing resulted in 64 positive diagnoses for LHBT pathology using the Speed test, 28 using the Yergason test, 37 using the Kibler test and 50 using the bicipital groove tenderness test. MRI observations found 10 positive LHBT pathology diagnoses according to signal intensity, 19 with regard to the diameter and 9 with regard to position. Intra‐operative arthroscopic findings, considered as gold standard, positively identified 43 pathological LHBT (Table 2).

**Table 2:** Individual diagnostic findings.

	Gold standard	Clinical tests				MRI evaluations		
	Arthroscopic findings	Speed	Yergason	Kibler	Pain on palpation	Signal anomaly	LHBT diameter	LHBT position
Positive diagnosis for LHBT pathology	43	64	28	37	50	10	19	9
Negative diagnosis for LHBT pathology	57	36	72	63	50	85	81	91
True positives		32	18	24	31	27	16	5
True negatives		25	47	44	38	23	54	53
False positives		32	10	13	19	32	3	4
False negatives		11	25	19	12	13	27	38

Abbreviations: LHBT, long head of the biceps tendon; MRI, magnetic resonance imaging.

The Speed's test had the highest sensitivity (0.74; 95% confidence interval [CI]: 0.59%–0.86%), followed by the bicipital groove tenderness test (0.72; 95% CI: 0.56%–0.85%) (Table 3).

**Table 3:** Individual diagnostic accuracy.

						Sensitivity					Specificity					Accuracy					PPV					NPV					Se + Sp
	N	TP	FP	FN	TN	Est.					Est.					Est.					Est.					Est.					
Clinical test																															
Speed	100	32	32	11	25	0.74				(0.59–0.86)	0.44				(0.31–0.58)	0.57				(0.47–0.67)	0.50				(0.37–0.63)	0.69				(0.52–0.84)	1.18
Yergason	100	18	10	25	47	0.42				(0.27–0.58)	0.82				(0.70–0.91)	0.65				(0.55–0.74)	0.64				(0.44–0.81)	0.65				(0.53–0.76)	1.24
Kibler	100	24	13	19	44	0.56				(0.40–0.71)	0.77				(0.64–0.87)	0.68				(0.58–0.77)	0.65				(0.47–0.80)	0.70				(0.57–0.81)	1.33
Tenderness	100	31	19	12	38	0.72				(0.56–0.85)	0.67				(0.53–0.79)	0.69				(0.59–0.78)	0.62				(0.47–0.75)	0.76				(0.62–0.87)	1.39
MRI observation																															
Signal	95	27	32	13	23	0.68				(0.51–0.81)	0.42				(0.29–0.56)	0.53				(0.42–0.63)	0.46				(0.33–0.59)	0.64				(0.46–0.79)	1.09
Diameter	100	16	3	27	54	0.37				(0.23–0.53)	0.95				(0.85–0.99)	0.70				(0.60–0.79)	0.84				(0.60–0.97)	0.67				(0.55–0.77)	1.32
Position	100	5	4	38	53	0.12				(0.04–0.25)	0.93				(0.83–0.98)	0.58				(0.68–0.68)	0.56				(0.21–0.86)	0.58				(0.47–0.68)	1.05

Abbreviations: Est., estimation; FN, false negative; FP, false positive; MRI, magnetic resonance imaging; N, cohort size; NPV, negative predictive value; PPV, positive predictive value; Se, sensitivity; Sp, specificity; TN, true negative; TP, true positive.

For MRI, the signal intensity had the highest sensitivity (0.68; 95% CI: 0.51%–0.81%), while both diameter and position had low sensitivity (0.37; 95% CI: 0.23%–0.53%; and 0.12; 95% CI: 0.04%–0.25%), but high specificity (0.95; 95% CI: 0.85%–0.99%; and 0.93; 95% CI: 0.83%–0.98%) (Table 3).

### Multiple criteria tests

A total of 29 combinations were tested to obtain the best diagnostic algorithm for LHBT pathologies. Only four combinations reached a sensitivity ≥0.75, but had a specificity <0.45 (Table 4). The ‘Speed or Signal’ combination achieved the highest sensitivity (Se: 0.88; 95% CI: 0.73%–0.96%; Sp: 0.20; 95% CI: 0.10%–0.33%), followed by the ‘Speed or Diameter’ combination (Se: 0.78; 95% CI: 0.63%–0.89%; Sp: 0.42; 95% CI: 0.29%–0.56%), the ‘Speed or Position or Diameter’ combination (Se: 0.77; 95% CI: 0.61%–0.88%; Sp: 0.40; 95% CI: 0.28%–0.54%), and finally the ‘Yergason or Signal or Position’ combination (Se: 0.75; 95% CI: 0.59%–0.87%; Sp: 0.33; 95% CI: 0.21%–0.47%). Of these combinations, the best balance of sensitivity and specificity was achieved for the ‘Speed or Diameter’ combination (Se + Sp, 1.20).

**Table 4:** Combined diagnostic accuracy.

						Sensitivity					Specificity					Accuracy					PPV					NPV					Se + Sp
	N	TP	FP	FN	TN	Est.					Est.					Est.					Est.					Est.					
Combination of two criteria																															
Speed AND Diameter	100	15	2	28	55	0.35				(0.21–0.51)	0.96				(0.88–1.00)	0.70				(0.60–0.79)	0.88				(0.64–0.99)	0.66				(0.55–0.76)	1.31
Speed OR Diameter	100	33	33	10	24	0.78				(0.63–0.89)	0.42				(0.29–0.56)	0.58				(0.48–0.68)	0.51				(0.39–0.64)	0.71				(0.53–0.85)	1.20
Speed AND Position	100	5	3	38	54	0.12				(0.04–0.25)	0.95				(0.85–0.99)	0.59				(0.49–0.69)	0.63				(0.24–0.91)	0.59				(0.48–0.69)	1.06
Speed OR Position	100	32	33	11	24	0.74				(0.59–0.86)	0.42				(0.29–0.56)	0.56				(0.46–0.66)	0.49				(0.37–0.62)	0.69				(0.51–0.83)	1.17
Speed AND Signal	95	21	18	19	37	0.53				(0.36–0.68)	0.67				(0.53–0.79)	0.61				(0.51–0.71)	0.54				(0.37–0.70)	0.66				(0.52–0.78)	1.20
Speed OR Signal	95	35	44	5	11	0.88				(0.73–0.96)	0.20				(0.10–0.33)	0.48				(0.38–0.59)	0.44				(0.33–0.56)	0.69				(0.41–0.89)	1.08
Yergason AND Diameter	100	11	2	32	55	0.26				(0.14–0.41)	0.96				(0.88–1.00)	0.66				(0.56–0.75)	0.85				(0.55–0.98)	0.63				(0.52–0.73)	1.22
Yergason OR Diameter	100	23	11	20	46	0.53				(0.38–0.69)	0.81				(0.68–0.90)	0.69				(0.59–0.78)	0.68				(0.49–0.83)	0.70				(0.57–0.80)	1.34
Yergason AND Position	100	3	1	40	56	0.07				(0.01–0.19)	0.98				(0.91–0.01)	0.59				(0.49–0.69)	0.75				(0.19–0.99)	0.58				(0.48–0.68)	1.05
Yergason OR Position	100	20	13	23	44	0.47				(0.31–0.62)	0.77				(0.64–0.87)	0.64				(0.54–0.73)	0.61				(0.42–0.77)	0.66				(0.53–0.77)	1.24
Yergason AND Signal	95	14	4	26	51	0.35				(0.21–0.52)	0.93				(0.82–0.98)	0.68				(0.58–0.78)	0.78				(0.52–0.94)	0.66				(0.55–0.77)	1.28
Yergason OR Signal	95	29	36	11	19	0.73				(0.56–0.85)	0.35				(0.22–0.49)	0.51				(0.40–0.61)	0.45				(0.32–0.57)	0.63				(0.44–0.80)	1.07
Speed AND Yergason	100	17	9	26	48	0.40				(0.25–0.56)	0.84				(0.72–0.93)	0.65				(0.55–0.74)	0.65				(0.44–0.83)	0.65				(0.53–0.76)	1.24
Speed AND Kibler	100	24	12	19	45	0.56				(0.40–0.71)	0.79				(0.66–0.89)	0.69				(0.59–0.78)	0.67				(0.49–0.81)	0.70				(0.58–0.81)	1.35
Speed AND Tenderness	100	28	17	15	40	0.65				(0.49–0.79)	0.70				(0.57–0.82)	0.68				(0.58–0.77)	0.62				(0.47–0.76)	0.73				(0.59–0.84)	1.35
Yergason AND Kibler	100	13	8	30	49	0.30				(0.17–0.46)	0.86				(0.74–0.94)	0.62				(0.52–0.72)	0.62				(0.38–0.82)	0.62				(0.50–0.73)	1.16
Yergason AND Tenderness	100	17	7	26	50	0.40				(0.25–0.56)	0.88				(0.76–0.95)	0.67				(0.57–0.76)	0.71				(0.49–0.87)	0.66				(0.54–0.76)	1.27
Kibler AND Tenderness	100	21	7	22	50	0.49				(0.33–0.65)	0.88				(0.76–0.95)	0.71				(0.61–0.80)	0.75				(0.55–0.89)	0.70				(0.57–0.80)	1.37
Combination of three criteria																															
Speed AND Position AND Diameter	100	4	0	39	57	0.09				(0.03–0.22)	1.00				(0.94–1.00)	0.61				(0.51–0.71)	1.00				(0.40–1.00)	0.59				(0.49–0.69)	1.09
Speed AND Position OR Diameter	100	17	6	26	51	0.40				(0.25–0.56)	0.89				(0.78–0.96)	0.68				(0.58–0.77)	0.74				(0.52–0.90)	0.66				(0.55–0.77)	1.29
Speed OR Position AND Diameter	100	15	2	28	55	0.35				(0.21–0.51)	0.96				(0.88–1.00)	0.70				(0.60–0.79)	0.88				(0.64–0.99)	0.66				(0.55–0.76)	1.31
Speed OR Position OR Diameter	100	33	34	10	23	0.77				(0.61–0.88)	0.40				(0.28–0.54)	0.56				(0.46–0.66)	0.49				(0.37–0.62)	0.70				(0.51–0.84)	1.17
Yergason OR Signal AND Position	95	4	3	36	52	0.10				(0.03–0.24)	0.95				(0.85–0.99)	0.59				(0.48–0.69)	0.57				(0.18–0.90)	0.59				(0.48–0.69)	1.05
Yergason OR Signal OR Position	95	30	37	10	18	0.75				(0.59–0.87)	0.33				(0.21–0.47)	0.51				(0.40–0.61)	0.45				(0.33–0.57)	0.64				(0.44–0.81)	1.08
Speed AND Yergason AND Kibler	100	13	8	30	49	0.30				(0.17–0.46)	0.86				(0.74–0.94)	0.62				(0.52–0.72)	0.62				(0.38–0.82)	0.62				(0.50–0.73)	1.16
Speed AND Yergason AND Tenderness	100	17	6	26	51	0.40				(0.25–0.56)	0.90				(0.78–0.96)	0.68				(0.58–0.77)	0.74				(0.52–0.90)	0.66				(0.55–0.77)	1.29
Speed AND Kibler AND Tenderness	100	21	7	22	50	0.49				(0.33–0.65)	0.88				(0.76–0.95)	0.71				(0.61–0.80)	0.75				(0.55–0.89)	0.70				(0.57–0.80)	1.37
Yergason AND Kibler AND Tenderness	100	13	5	30	52	0.30				(0.17–0.46)	0.91				(0.81–0.97)	0.65				(0.55–0.74)	0.72				(0.47–0.90)	0.63				(0.52–0.74)	1.22
Combination of four criteria																															
Speed AND Yergason AND Kibler AND Tenderness	100	13	5	30	52	0.30				(0.17–0.46)	0.91				(0.81–0.97)	0.65				(0.55–0.74)	0.72				(0.47–0.90)	0.63				(0.52–0.74)	1.22

Abbreviations: Est., estimation; FN, false negative; FP, false positive; MRI, magnetic resonance imaging; N, cohort size; NPV, negative predictive value; PPV, positive predictive value; Se, sensitivity; Sp, specificity; TN, true negative; TP, true positive.

## DISCUSSION

The most important findings of this study were that, for the diagnosis of LHBT pathology the combination of ‘Speed test or Signal intensity’ substantially improved the sensitivity (Se, 0.88) but yielded the lowest specificity (Sp, 0.20). These findings confirm the hypothesis that a combination of MRI observations and clinical tests grants higher sensitivity compared to MRI observations alone or clinical tests alone. The clinical relevance of these findings is that using the combination ‘Speed or Signal’ for preoperative diagnosis, 88% of pathologic LHBTs would be correctly diagnosed, while 80% of healthy LHBTs could be misdiagnosed as pathologic.

The best clinical test for preoperative assessment of the state of the LHBT has been previously investigated, and a number of tests have been created for the shoulder or the biceps. Holtby et al. [20] reported diagnostic values for the Speed and Yergason tests, and found a sensitivity of 0.32 and 0.43, respectively. In 2007, Gill et al. [15] investigated the diagnostic values of 10 clinical tests for LHBT tear, and found sensitivity values ranging from 0.17 to 0.68, the highest being for the active compression palm down test. The diagnostic values reported in the studies are insufficient for reliable preoperative diagnosis of LHBT pathology. In a systematic review by Rosas et al. [25], the authors developed a practical, evidence‐based clinical examination algorithm to accurately diagnose patients with LHBT pathology. Rosas et al. found the highest sensitivity (Se, 0.88) by combining bicipital groove tenderness (Se, 0.57) with the uppercut test (Se, 0.73). In contrast, the present study found sensitivities ranging from 0.42 to 0.74 for individual clinical tests, while the combination that provided the highest sensitivity of 0.65 was obtained when using ‘Speed and Tenderness’. The present study indicates that combining clinical tests only does not provide adequate sensitivity for the assessment of LHBT pathology.

As for clinical tests, the sensitivity of MRI findings has been investigated in the literature. In 2019, Kim et al. [22] assessed the diagnostic value for MRI findings of abnormal signs (diameter, contour irregularity, and signal alteration), which resulted in a sensitivity of 0.52–0.67 in the parasagittal view, and 0.58–0.67 in the axial view. Shibayama et al. [27] investigated LHBT diameter and change in signal intensity as diagnostic criteria using MRI and found a higher sensitivity using diameter (Se, 0.84) compared to change in signal intensity (Se, 0.52). Finally, in a meta‐analysis by Lalevée et al. [23], the sensitivity of MRI for diagnosis of LHBT pathologies was investigated by type of pathology. Lalevée et al. [23] reported a pooled sensitivity of 0.56 for full‐thickness LHBT tears, 0.52 for partial‐thickness LHBT tears and 0.58 for any LHBT tear. In the present study, the sensitivity of individual MRI observations ranged from 0.12 to 0.68. Individual MRI findings seemingly have low to moderate sensitivity in diagnosing any type of LHBT lesion, while the combination of MRI findings and clinical tests substantially improved the sensitivity (Se, 0.88).

Lesions of the LHBT are a common cause of pain and dysfunction in shoulders with RCTs, and early diagnosis and treatment can prevent further degeneration of the LHBT [6, 9, 10, 23, 24]. The most common treatment options are tenotomy or tenodesis [5, 18]. In 2005, Walch et al. [29] reported that spontaneous rupture of the LHBT during the evolution of RCTs resulted in pain relief, and henceforth popularised systematic tenodesis or tenotomy of the LHBT during RCR [18], even when the LHBT showed no macroscopic signs of pathology. While satisfactory outcomes have been reported for both tenodesis and tenotomy, there is yet no consensus regarding the best treatment, as both can lead to complications [1, 28]. Tenotomy is often associated with the occurrence of a Popeye sign and a decrease in strength, while tenodesis requires longer recovery time, and can result in cramps [5, 14, 18]. There is a general consensus, however, that tenotomy or tenodesis is necessary if the LHBT is pathologic during RCR, to avoid residual bicipital pain and the need for reoperation, which is why diagnosis requires high sensitivity [15, 20, 22, 23, 25, 27].

The present study revealed that a combination of clinical tests and MRI findings (Se, 0.88) resulted in a higher sensitivity than clinical tests (Se, 0.74) or MRI findings alone (Se, 0.68). Correct preoperative assessment is important, as it allows to manage patients' expectations and intra‐operative time. Nonetheless, intra‐operative arthroscopic findings could serve as the final assessment of the intra‐articular portion of LHBT, during which a pathologic LHBT that was misdiagnosed as healthy, would still be correctly treated. To the author's knowledge, no other study has investigated the combination of clinical tests and MRI to detect LHBT pathologies. The present study, however, found a sensitivity of 0.88 by using the ‘Speed or Signal’. In a meta‐analysis by Courage et al. [11], comparing diagnostic values of clinical tests versus ultrasound findings, the pooled sensitivity of ultrasound assessment of LHBT was 0.70, which is more than what was found in the literature, as well as in the present study for MRI assessment. Even though the present study found a high sensitivity, it is possible that future studies using other imaging modalities or combinations thereof, find even higher sensitivities. Future studies should evaluate the reliability of ultrasound, as an alternative to MRI, to maximise sensitivity of diagnosis of LHBT pathologies, as ultrasound is considerably faster and cheaper to perform, and therefore could facilitate decision‐making during initial patient assessment.

The findings of the present study should be interpreted with the following limitations in mind. First, the number of patients excluded for missing MRI assessment was approximately 40%, as some patients had computed tomography arthrography (CTA) instead of MRI. Second, for five patients, signal intensity was not measured on MRI, which could leave doubts on the reliability of this single criterion, and its use as diagnostic criteria. Third, as some MRI criteria can be subjective to interpret, it is possible that MRI readings might have differed between the eight surgeons since interobserver repeatability was not assessed, and the ultimate diagnostic repeatability of the combinations used cannot be ascertained, although the authors had sufficient confidence in the repeatability of MRI as reported in numerous previous studies [22, 27]. Finally, the present study is based on MRI assessments by surgeons, which could differ from assessments by radiologists. It is worth noting, however, that the eight surgeons had agreed on common assessment criteria, while the undetermined number of radiologists could have increased heterogeneity. Nonetheless, the strength of the present study is that it is the first to investigate the combination of clinical tests and MRI observations for the diagnosis of LHBT pathology, which could provide greater diagnostic value for clinicians.

## CONCLUSION

For the diagnosis of LHBT pathologies, the combination ‘Speed or Signal’ could diagnose 88% of pathologic LHBTs correctly, while 80% of healthy LHBTs could be misdiagnosed as pathologic.

## AUTHOR CONTRIBUTIONS

**David Gallinet** and **Jacques Guery**: Funding acquisition; data collection; methodology; supervision; validation. **Maxime Antoni**, **Julien Berhouet** and **Christophe Charousset**: Data collection; methodology; validation. **Chinyelum Agu**, **Floris van Rooij** and **Mo Saffarini**: Methodology; manuscript writing; formal analysis; validation.

## CONFLICT OF INTEREST STATEMENT

David Gallinet reports consulting and royalties from moveUP outside the submitted work. Maxime Antoni reports fees from ConMed and fees and royalties FX Shoulder Solutions outside the submitted work. Julien Berhouet reports consulting for Wright Medical outside the submitted work. Jacques Guery reports fees from moveUP outside of the submitted work. The remaining authors declare no conflict of interest.

## ETHICS STATEMENT

All patients provided informed consent and the study was approved by the local ethics committee (IRB: 2018‐A01382‐53).

## Data Availability

All data are available upon reasonable request.
